# Increase in temperature enriches heat tolerant taxa in *Aedes aegypti* midguts

**DOI:** 10.1038/s41598-020-76188-x

**Published:** 2020-11-05

**Authors:** Gorreti Maria Onyango, M. Sean Bialosuknia, F. Anne Payne, Nicholas Mathias, T. Alexander Ciota, D. Laura Kramer

**Affiliations:** 1 Wadsworth Center, New York State Department of Health, 5668 State Farm Road, Slingerlands, NY 12159 USA; 2 School of Public Health, State University of New York Albany, 1400 Washington Avenue, Albany, NY 12222 USA

**Keywords:** Microbiology, Microbial communities

## Abstract

Insect midgut microbial symbionts have been considered as an integral component in thermal adaptation due to their differential thermal sensitivity. Altered midgut microbial communities can influence both insect physiology and competence for important vector-borne pathogens. This study sought to gain insights into how *Aedes aegypti* midgut microbes and life history traits are affected by increase in baseline diurnal temperature. Increase in temperature resulted in the enrichment of specific taxa with *Bacillus* being the most enriched. *Bacillus* is known to be heat tolerant. It also resulted in a dissimilar microbial assemblage (Bray–Curtis Index, PERMANOVA, F = 2.2063; R^2^ = 0.16706; *P* = 0.002) and reduced survivorship (Log-rank [Mantel-Cox] test, Chi-square = 35.66 df = 5, *P* < 0.0001). Blood meal intake resulted in proliferation of pathogenic bacteria such as *Elizabethkingia* in the midgut of the mosquitoes. These results suggest that alteration of temperature within realistic parameters such as 2 °C for *Ae. aegypti* in nature may impact the midgut microbiome favoring specific taxa that could alter mosquito fitness, adaptation and vector–pathogen interactions.

## Introduction

The global mean surface temperature is predicted to rise by 1.6–6.6 °C in the late twenty-first Century^1^. Physiology^2–4^, behavior^5^, ecology^6,7^ and evolutionary history^8–10^ may influence the biological responses of organisms to global climate change. *Aedes aegypti* is the vector of both Zika and dengue viruses which are of global health importance. Dengue fever has spread to five continents resulting in over 3 billion people at risk of contracting the disease^11^ while emergent Zika outbreaks have resulted in a public health emergencies of international concern.

Recently, it has become clear that microbial symbionts often facilitate or limit adaptation to environmental fluctuations^12^. Hence, species and communities’ response to climate change may be significantly impacted by the adaptability of their microbial partners. Insect midguts provide a receptive environment for microbial colonization and the midgut bacteria play a myriad of important roles in the survival of their host^13–15^. Midgut bacteria of insects have demonstrated properties including: nutrition, protection from parasites and pathogens, modulation of immune responses, mating and communication^16^.

It is therefore important to understand how the microbes of medically important vectors such as *Aedes* mosquitoes will react to increase in temperature as this may provide insights into how this vector will change in response to climate variation. By extension, this will allow the projection into how the landscape of the diseases of global importance caused by the pathogens they transmit will be altered.

The impact of temperature change on the midgut microbiome diversity of insects has mainly been studied with constant temperature. The results of such studies show a significant reduction in the richness of the midgut microbiome to complete loss of midgut microbiome as well as reduction in the vertical transmission to the next generation^17–20^. Given that temperatures fluctuate in nature; using constant temperature to study the impact of temperature on the midgut microbiome may not provide an accurate depiction. This gap in knowledge is highly important given the well documented role of the midgut microbiome in vectorial capacity^21–28^. By adjusting the baseline temperature to the future predicted increase in temperature, we studied the life history traits and midgut microbiome of *Ae. aegypti* from two geographical locations. We tested the hypothesis that significant variation in composition of *Ae. aegypti* midgut microbiome is associated with increased temperature. The aims of this study were: to delineate the impact of increase in diurnal temperature on midgut microbiome composition and mosquito life history traits; to describe the effect of non-infectious blood meal intake on the midgut microbial diversity and finally to examine population differences in microbial assemblage, comparing two *Ae. aegypti* populations.

## Results

### Temperature increase impacts the immature stages and adult emergence of *Aedes* in a population-specific manner

The immature stages of the mosquitoes were reared in three replicates which were statistically similar, hence all the calculations on the impact of temperature on the immature stages as well as adult emergence were based on mean values of the biological replicates. Increase in temperature had a population-specific impact on the egg eclosion rates and larval death rates. The egg eclosion rates of the Miami population improved with an increase in temperature (Fisher’s exact test *P* < 0.0001) (Table 1); the converse was observed in the Mexico population as shown by a reduction in their egg eclosion rates when the temperature was increased (Fisher’s exact test *P* < 0.0001) (Table 1). Between the two populations at the L temperature regime, AEG MX had a higher egg eclosion rate than AEG MI (Chi-square = 31.52, df = 1, *P* < 0.0001) (Table 1). In contrast, egg eclosion rate was higher for the AEG MI population as compared to the AEG MX population at the H temperature regime (Chi-square = 242.5, df = 1, *P* < 0.0001) (Table 1). Lower larval mortality rates among the AEG MI population were measured at higher temperatures (Fisher’s exact test *P* < 0.0001) (Table 1), while in the AEGMX population, increased temperature resulted in higher mortality rates of larvae (Fisher’s exact test *P* = 0.02) (Table 1). The mortality rates of larvae were higher among the AEGMX population at the H temperature regime relative to the AEGMI population (Chi-square = 29.81, df = 1, *P* < 0.0001) (Table 1), however no difference in larval mortality rates were measured between populations at the L temperature regime (Chi-square = 2.320, df = 1, *P* = 0.1277) (Table 1).

**Table 1:** Rates of life history traits.

	AEG MI H (CV)	AEG MI L (CV)	AEG MX H (CV)	AEG MX L (CV)
Egg eclosion rates (%)	77 (0.01)	56 (0.07)	46 (0.25)	67(0.19)
Larval death rates (%)	0.48 (1.96)	3 (0.24)	4.39 (0.19)	1.88 (0.47)
Pupation rates (%)	48 (0)	68 (0)	66 (0)	79 (0)
Blood feeding rates (%)	26	21	30	31
Eggs deposited (Average)	1072 (0.39)	943 (0.42)	1084 (0.48)	1086 (0.50)

*CV* coefficient of variation.

Increase in temperature reduced the pupation rates across both populations [AEGMI (Fisher’s exact test *P* < 0.0001)] [AEGMX (Fisher’s exact test *P* < 0.0001)] (Table 1) yet, this effect was larger for the AEGMI population (Chi-square = 38.53, df = 1, *P* < 0.0001) (Table 1). At the L temperature regime, the AEG MX population had a higher pupation rate compared to the AEG MI population (Chi-square = 22.42, df = 1, *P* < 0.0001) (Table 1).

**Figure 1:**
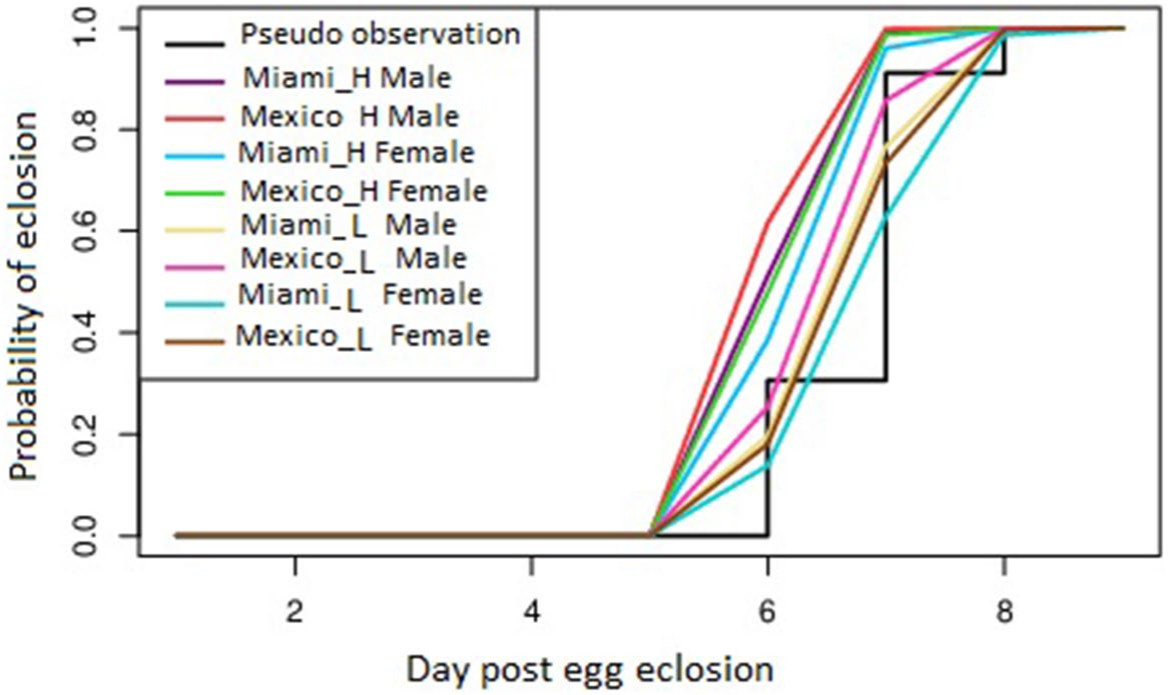
Cox proportional hazard model on adult emergence rates across temperature regimes. *Ae. aegypti* mosquitoes were collected from Miami and Mexico and reared at two different temperature regimen, baseline temperature L (D30N26) and elevated temperature H (D32N28). A cox proportional hazard model to evaluate the effect of population, temperature and sex was designed. Temperature, location and sex are significant factors that affected eclosion over time. However, there was no interaction between temperature and Location as variables. The X axis represents the days post egg eclosion while Y axis represents the probability of eclosion.

A cox proportional hazard model was computed using R, in order to evaluate the impact of temperature, population and sex, as well as the interaction among these variables. Temperature, population and sex all significantly affected eclosion over time. However, there was no interaction between temperature and population (Supplementary file no. 1), (Fig. 1).

The majority of adults reared at the H temperature regime emerged on day 7 (post egg eclosion), as compared to day 8 (post egg eclosion) for the L regime. In all populations, the total number of adults emerging were calculated, and the adult males emerged prior to females. AEGMI H males: 77% emerged on day 7 and 23% on day 8; AEGMI H females: 43% emerged on day 7 and 57% on day 8. AEGMX H males: 77% emerged on day 7 and 23% on day 8; AEGMX H females: 63% emerged on day 7 and 37% on day 8. There were no differences between the populations in the emergence rates (F test *P* = 0.9205).

It is noteworthy that, more than 50% of the AEGMX L females emerged on the first day when the population recorded its highest adult emergence ratio (77% male and 63% female) (Chi-square = 0.2488, df = 1, *P* = 0.6179) as compared to the AEGMI L (77% male and 43% female) Chi-square = 16.32, df = 1, *P* < 0.0001).

### Increase in temperature reduces the wing length and adult survivorship

**Figure 2:**
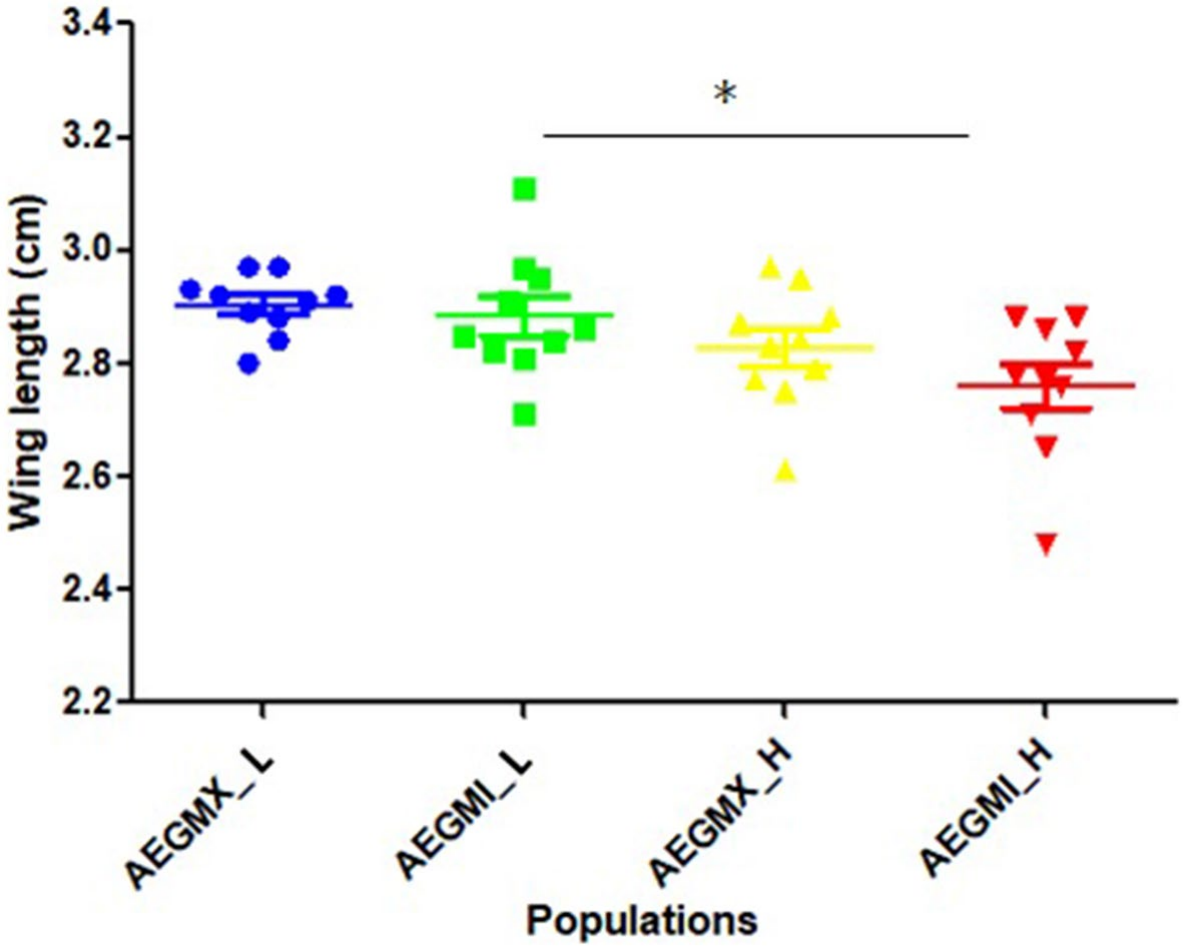
Impact of increased temperature on wing length of *Ae. aegypti*. The impact of increased temperature on the body size was determined by measuring the wing length of the female mosquitoes. Both the Miami and Mexico *Ae. aegypti* female adults reared at the H temperature regime demonstrated a significantly reduced wing length as compared to the L temperature regime [Kruskal–Wallis statistic = 10.72; *P* (0.0133)].

**Figure 3:**
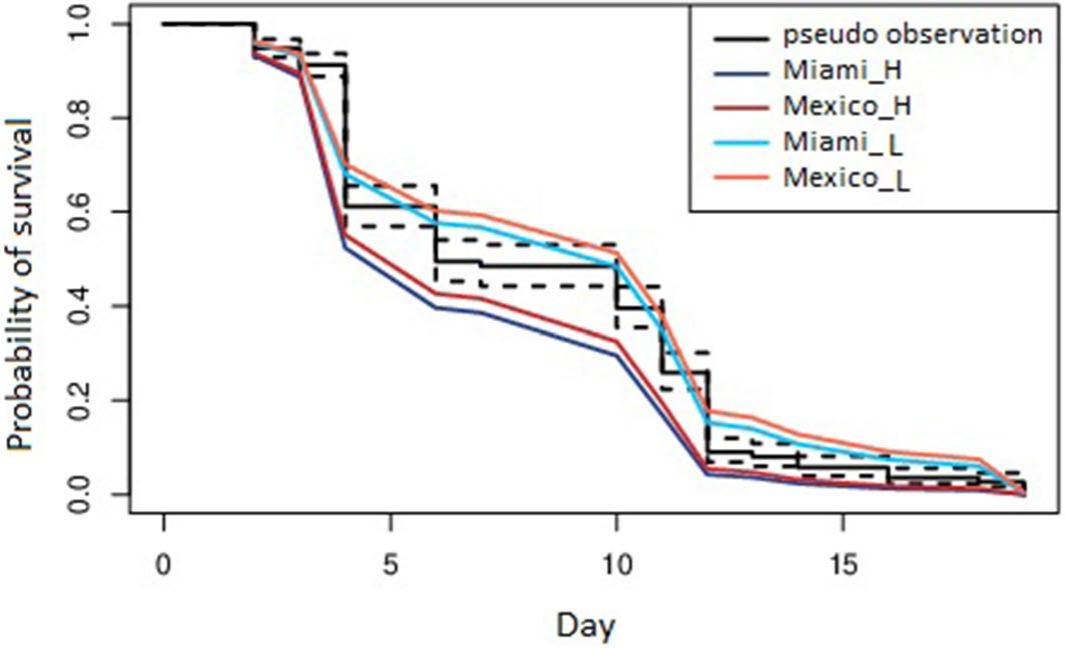
The effect of temperature on longevity of *Ae. aegypti*. The impact of temperature on longevity of the mosquitoes was measured by counting the number of dead adult mosquitoes from the first day of emergence. Increased temperature significantly reduced the longevity of both populations of *Ae. aegypti* adults (Log-rank [Mantel-Cox] test, Chi-square = 35.66, df = 5, *P* < 0.0001). A cox proportional hazard model to test the influence of geographical location and temperature as variable on survivability demonstrated that temperature as a variable influenced survivability but location did not influence the model.

The adults reared at the L temperature regime had a significantly longer wing length than the individuals reared at the H temperature regime [ANOVA, Kruskal–Wallis statistic = 10.72; *P* (0.0133)] (Fig. 2). A cox proportion hazard model demonstrated that temperature as a variable influenced longevity, however, population did not (Supplementary file 2), (Fig. 3).

The *Ae. aegypti* populations reared at the H temperature regime had a reduced longevity compared to the L temperature regime (Log-rank [Mantel-Cox] test, Chi-square = 35.66, df = 5, *P* < 0.0001)^29^ (Fig. 3). The survival to adulthood was inversely related to temperature [AEGMI (H—41%, L—56%); AEGMX (H—50%, L—78%)].

### Skip oviposition behavior is evident across temperature regime and populations

Skip oviposition behavior describes a behavior of *Ae. aegypti* and *Ae. albopictus* where they skip from oviposition site to oviposition site to distribute their eggs. The skip oviposition behavior has been observed in some natural populations of *Aedes* mosquitoes whereby eggs are deposited in more than one oviposition site. Skip oviposition behavior was evident in both temperature regimes of the AEG MI populations.

Increase in temperature did not affect the blood feeding rates of either population [AEGMI (Fisher’s exact test *P* = 0.0887)], [AEGMX (Fisher’s exact test *P* = 0.9205)] nor the egg oviposition rates [Kruskal–Wallis test *P* (0.3916)] (Table 1).

### Microbial profiles identified in *Aedes* midguts

**Figure 4:**
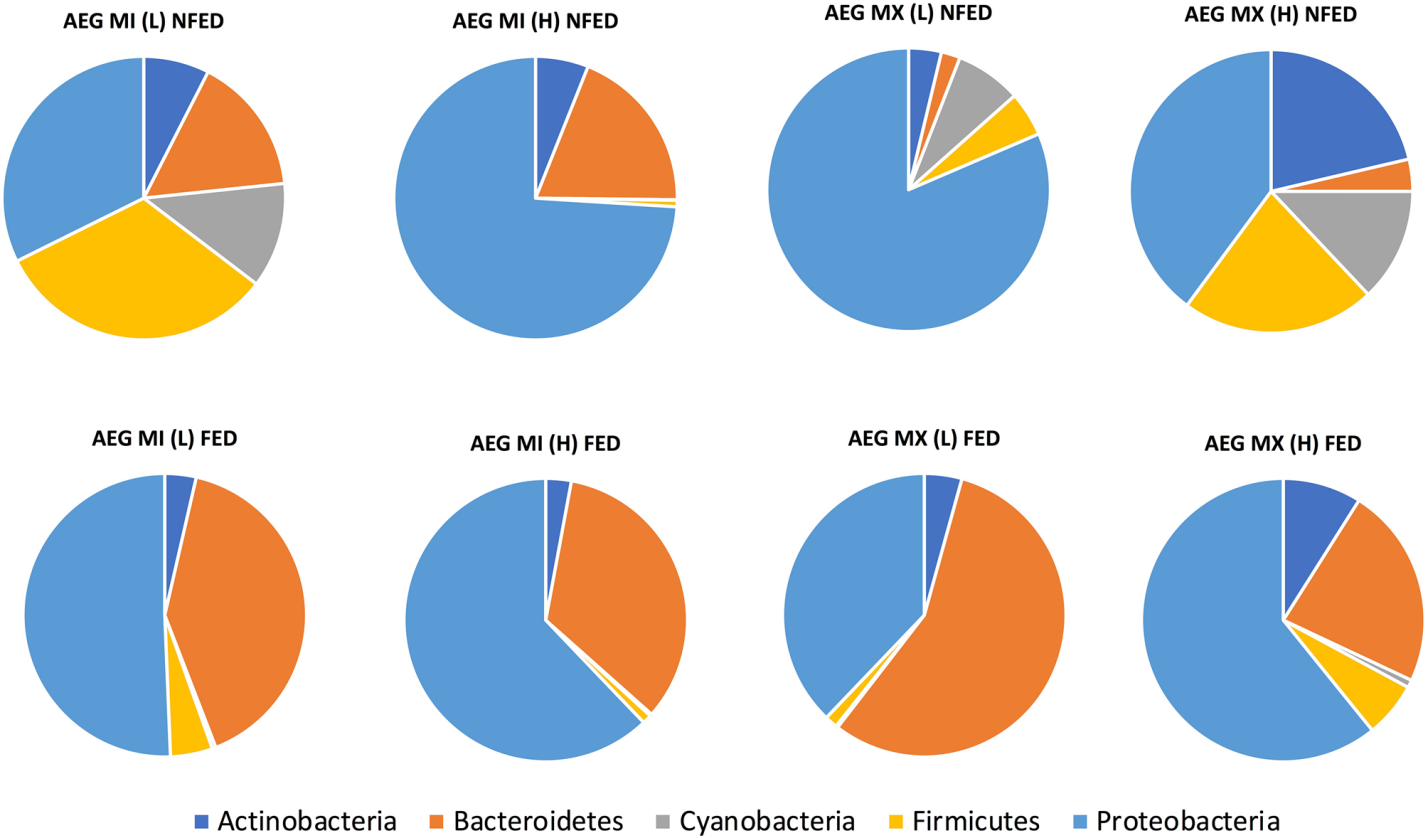
Relative richness (%) of the core phylotypes identified in *Ae. aegypti* midguts. The core gut microbiome phyla of *Ae. aegypti* was Actinobacteria, Bacteroidetes, Cyanobacteria, Firmicutes and Proteobacteria, respectively. Blood meal acquisition skewed the core phylotypes across the two populations (Bray–Curtis Index, PERMANOVA, F = 2.7182, R^2^ = 0.072067; *P* = 0.004).

To elucidate the impact of increase in temperature, population and blood meal intake on the midgut microbial profile, we sampled *Ae. aegypti* from both populations as well as temperature treatments. Total genomic DNA was extracted from adult female *Ae. aegypti* midguts two days post non-infectious blood meal. As a control, *16S rDNA* amplicons were obtained from midguts of adult female fed entirely on 10% sucrose. The sequencing effort generated a total of 6,925,549 reads with a maximum read length of 301 and a mean of 297. After cleaning and trimming the reads, a total of 4,275,936 reads were retained and the frequency of the reads ranged from 25,348–457,857 across the 40 samples analyzed. A total of 3116 OTU were identified. To reduce the potential of alpha and beta diversity biases, the OTU data was rarefied to a sample depth of 34,000 reads per sample. For bacterial profiling, three samples were eliminated from the study as they were below the selected 34,000 reads per sample that all the samples were rarefied to. The OTUs isolated in this study belonged to 5 core phyla: Proteobacteria, Bacteroidetes, Actinobacteria, Cyanobacteria and Firmicutes (Fig. 4).

### Temperature significantly impact midgut microbial communities

**Figure 5:**
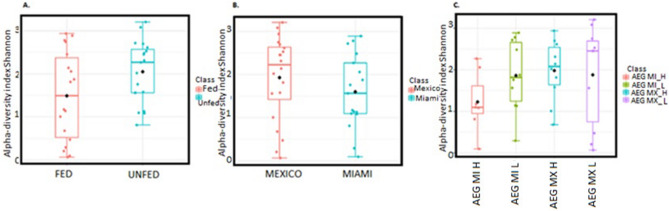
Calculation of the intra-diversity of the gut microbiome of the *Aedes* mosquitoes. Box plot representation of the diversity of bacterial taxa in *Ae. aegypti*. (**A**). Intake of a blood meal resulted in a homogenous microbial midgut both in terms of the richness and evenness [Alpha diversity, Shannon diversity Index *P* (0.067603; T-test statistic: − 1.8962]. (**B**) The midgut microbial richness and evenness within each population in Miami and Mexico were homogenous (Alpha diversity Shannon diversity Index *P* = 0.28213; T-test statistic: 1.0924) and (**C**) Increase in temperature did not alter the homogeneity in midgut microbiota across the two populations [Alpha diversity Shannon diversity Index *P* (0.37668) ANOVA F-value: 1.0663]. The X axis represents the combination of the populations and the temperature regimen that they were reared at. The abbreviation of the *Aedes aegypti* (AEG) population and the temperature regimen that they represent. Such that AEG MI H represents the *Ae. aegypti* collected from Miami (MI) and reared at the H temperature regimen, AEG MI M- *Ae. aegypti* collected from Miami (MI) and reared at the L temperature regimen, AEG MX H represents the *Ae. aegypti* collected from Mexico (MX) and reared at the H temperature regimen while AEG MX L represents the *Ae. aegypti* collected from Mexico (MX) and reared at the L temperature regimen.

**Figure 6:**
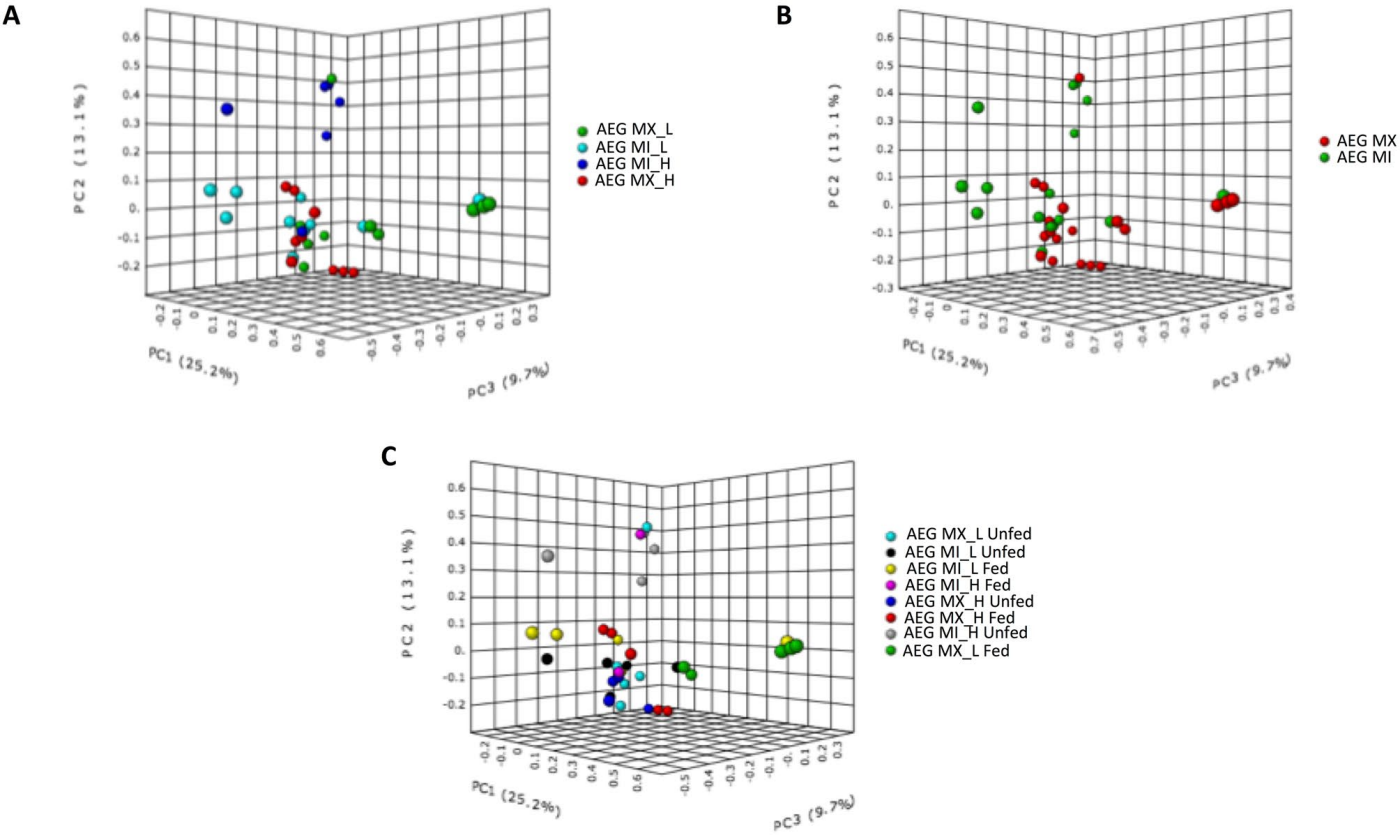
Comparison of bacterial community structure of *Ae aegypti* mosquitoes. A two-dimensional plot of the Bray–Curtis index. (**A**) Increase in temperature resulted in significant dissimilarity in the midgut microbial community between the H and L temperature regimen (Bray–Curtis Index, PERMANOVA, F = 2.2063; R^2^ = 0.16706; *P* = 0.002). (**B**) Phylogenetic differences between the two populations (Miami and Mexico) in the bacterial community assemblages was not observed {[PERMANOVA] F- value: 1.4023; R-squared: 0.038523; *P* (< 0.169)}. (**C**) Intake of a blood meal altered the microbial community in the midgut of *Ae. aegypti* individuals (Bray–Curtis Index, PERMANOVA, F = 2.7182, R^2^ = 0.072067; *P* = 0.004).

The midgut microbial abundance and evenness within each population in Miami and Mexico were homogenous in this study (Alpha diversity Shannon diversity Index *P* = 0.28213; T-test statistic: 1.0924) (Fig. 5B). Increase in temperature did not result in variation in richness or evenness among the individual midguts reared at H and L temperature regimen [Alpha diversity Shannon diversity Index *P* (0.37668) ANOVA F-value: 1.0663] (Fig. 5C), yet a significant dissimilarity in the midgut microbial community was identified between individuals reared at the H and L temperature regimen (Bray–Curtis Index, PERMANOVA, F = 2.2102; R^2^ = 0.16731; *P* = 0.003) (Fig. 6A).

Evenness in phyla distribution of the core microbiota was evident in the AEG MI (L) UNFED individuals [Proteobacteria (0.323), Bacteroidetes (0.158), Actinobacteria (0.075), Cyanobacteria (0.120) and Firmicutes (0.323)] and AEG MX (H) UNFED [Proteobacteria (0.399), Bacteroidetes (0.037), Actinobacteria (0.213), Cyanobacteria (0.130) and Firmicutes (0.222)]. However, AEG MI (H) UNFED [Proteobacteria (0.740) Bacteroidetes (0.191), Actinobacteria (0.060), Cyanobacteria (0.0006) and Firmicutes (0.007)] and AEG MX (L) UNFED [Proteobacteria (0.815), Bacteroidetes (0.022), Actinobacteria (0.037), Cyanobacteria (0.075) and Firmicutes (0.051)] had a skewed core microbiota dominated mainly by Proteobacteria (Fig. 4).

Measures of β-diversity to assess patterns in bacterial community assemblages demonstrated that there are not significant differences between the two populations (Bray–Curtis Index, PERMANOVA, F-value: 1.4001; R-squared: 0.038464; *P* = 0.169) (Fig. 6B).

**Figure 7:**
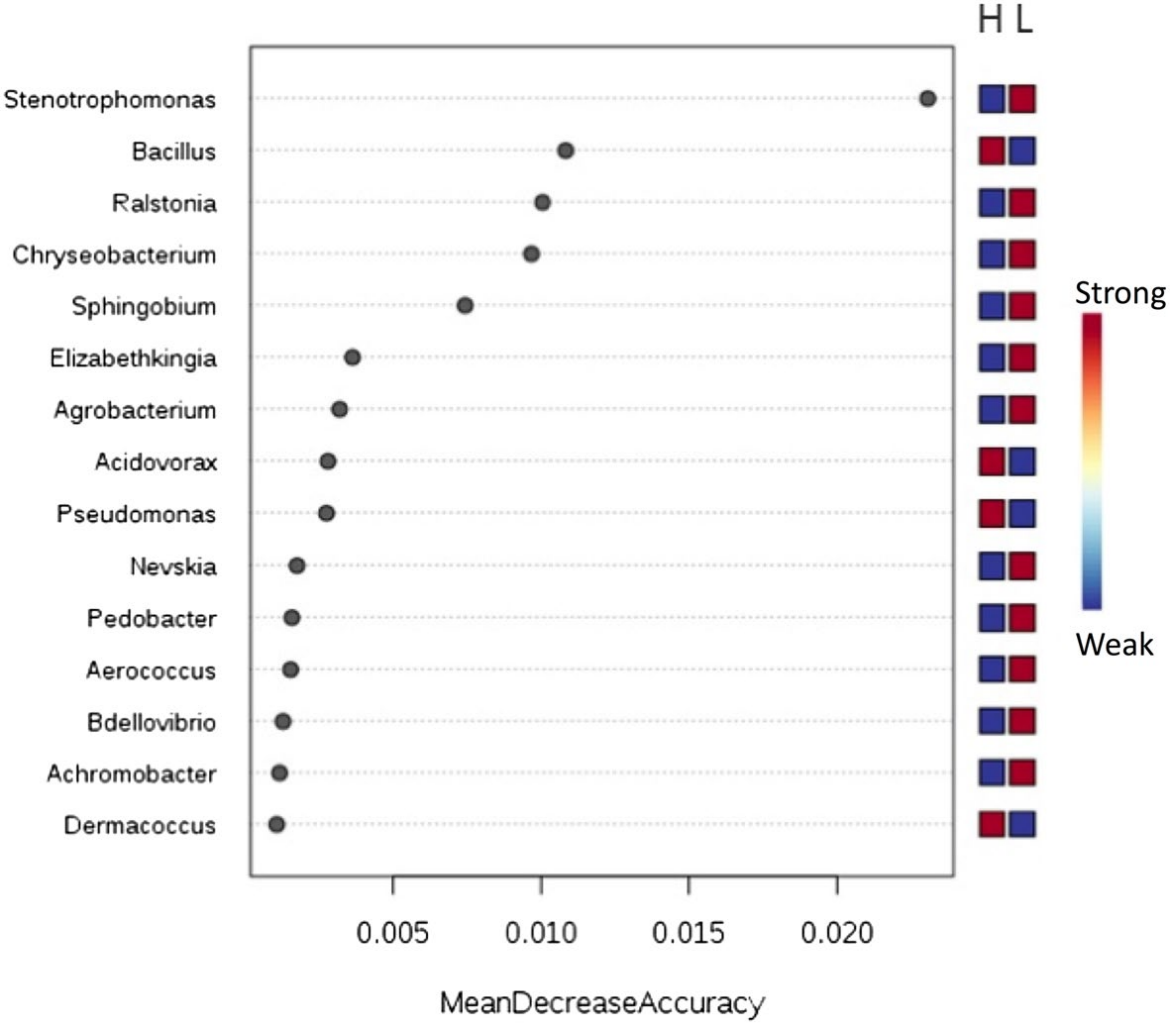
Enrichment of individual bacterial taxa is associated with a temperature increase in *Ae. Aegypti*. The Random forest classifier was used to determine the importance of differentially abundant taxa enriched due to increased temperature across both populations and feeding status using the random forest algorithm within Microbiome Analyst. A supervised classification algorithm of trees was created by using bootstrap samples while training data and random feature selection in tree induction. It is an ensemble of unpruned classification or regression trees trained with the bagging method. The Mean Decrease Accuracy obtained by removing the relationship of a taxa and measuring increase in error is reported for each taxon. The taxa with the highest mean decrease in accuracy is considered to have the highest association with the state. Taxa were listed in order of the strength of their level of association with temperature. *Stenotrophomonas* is highly associated with L regime while *Bacillus* is strongly associated with H temperature regime. L represents the D30N26 and H represents D32N28 temperature regime. As a taxon approaches red, it indicates richness while the closer it is to blue, the lower its richness in its representation in either H or L temperature regimes.

**Figure 8:**
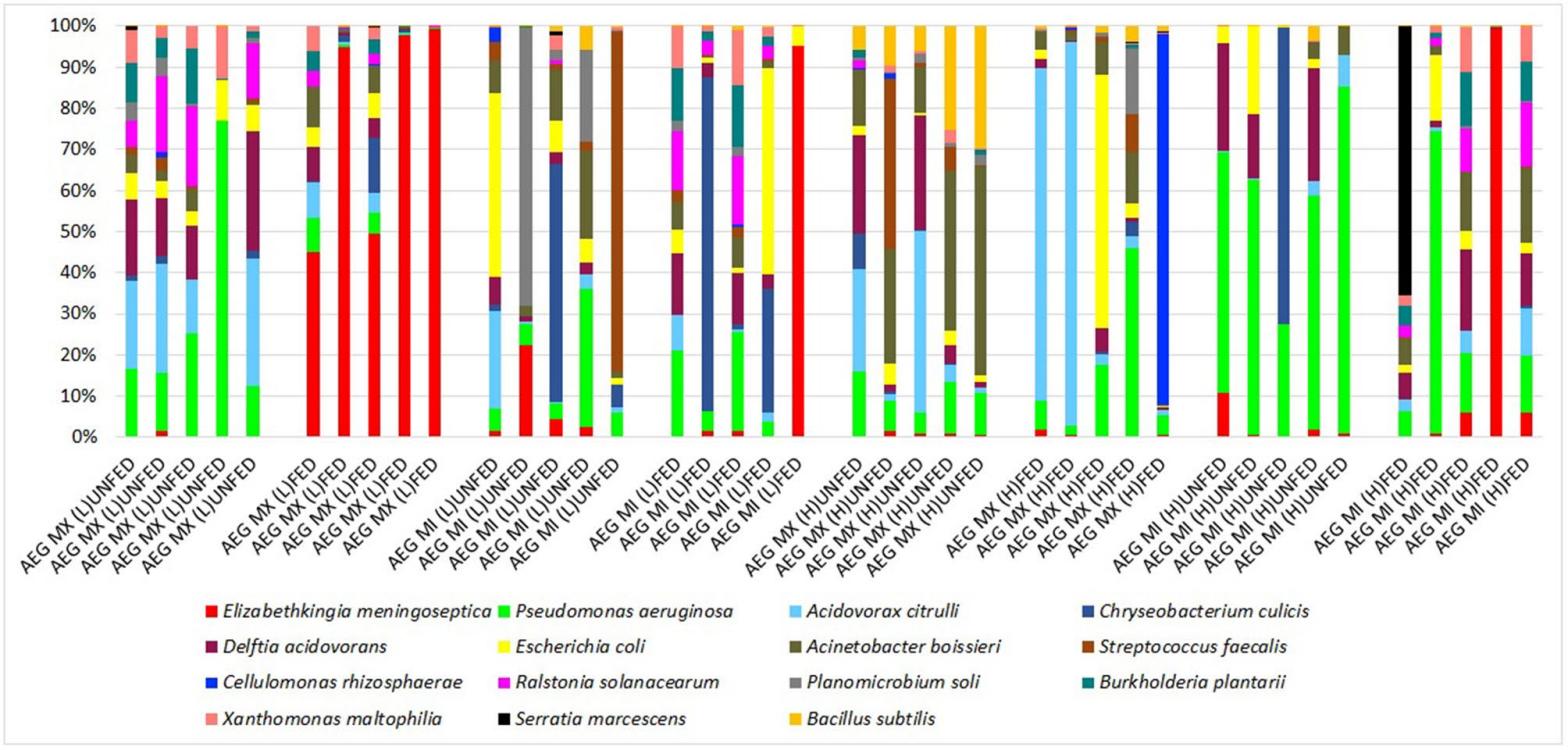
Bar plot representation of the taxa identified in *Ae. aegypti* population. A bar plot representation of relative richness of taxa in individual midguts across the different populations and blood meal intake status. The taxa include in the bar plot contained ≥ 10,000 sequence.

Increased temperature resulted in richness of *Bacillus subtilis*; the richness was notable among the AEG MX_H population (Fig. 7) (Supplementary File 3). *Acidovorax citrulli* and *Pseudomonas aeruginosa* were also enriched by increase in temperature and *Pseudomonas aeruginosa* was most enriched among the AEG MI_H followed by AEG MX_H (Fig. 8) while *Dermacoccus* was the least enriched of the taxa (Fig. 7). Previous work in our lab has demonstrated that *Elizabethkingia* thrives better in lower temperature than higher temperatures and this was evident in this study. *Stenotrophomonas* was highly associated with L regime (Fig. 7).

### Blood meal intake did not affect the richness of the midgut microbes but resulted in a heterogenous microbial community in the Ae. aegypti midgut

Blood meal intake significantly altered the homogeneity of the microbial community in the midgut of *Ae. aegypti* individuals (Bray–Curtis Index, PERMANOVA, F = 2.3422, R^2^ = 0.36116; *P* = 0.001) (Fig. 6C). However, the blood meal did not impact the richness and evenness of the midgut microbes [Alpha diversity, Shannon diversity Index *P* = 0.067603; T-test statistic: − 1.8962] (Fig. 5A). Blood meal ingestion resulted in the gut phyla being dominated mainly by Proteobacteria in both temperature regimes and populations (Fig. 4). The exception was AEG MX (L) gut microbial taxa, which shifted from richness with Proteobacteria (*Pseudomonas, Comamonadaceae, Ralstonia* and *Delftia*) (before blood meal ingestion) to Bacteroidetes (*Elizabethkingia*) (after blood meal) (Fig. 8). Another notable effect of blood meal ingestion was a reduction in the relative richness of Firmicutes (Fig. 4). Taxa for which richness increased significantly following blood feeding have been shown to demonstrate hemolytic activity, including *Elizabethkingia*^30^, *Pseudomonas*^31^ and *Enterobacteriaceae*^32^.

With the exception of AEG MX (L) FED (F) individuals, which demonstrated richness of *Elizabethkingia* taxa across individuals, variability in dominant taxa at the individual level was identified across groups: (AEG MI (L) F- *Elizabethkingia*, *Chryseobacterium* or *Enterobacteriaceae*; AEG MI (H) F- *Ralstonia*, *Pseudomonas* or *Elizabethkingia*; AEG MX (H) FED – *Comamonadaceae*, *Enterobacteriaceae* or *Streptococcus*) (Fig. 8).

## Discussion

This study has demonstrated selective enrichment of heat tolerant taxa (*Bacillus*) due to temperature increase. Increase in temperature due to climate change is expected to occur in the future^33,34^. *Aedes* mosquitoes vector several clinically important viruses, such as Zika virus, chikungunya virus, yellow fever virus and dengue virus^35^. The microbial community of the mosquito midgut plays a key role in determining the outcome of pathogen infection of mosquitoes^22–24^. Studying the impact of temperature increase on the diversity and function of gut microbial communities is important as it will result in insights on how ectothermic organisms will adapt to climate change in the future^36^. Studies have shown the activation of heat shock genes due to environmental stress of *Bacillus* spp^37,38^. A study with *Bacillus* spp. SUBB01 at high temperatures estimated that this species could survive at temperatures up to 53 °C^39^. Further, extracellular fractions of *Bacillus* spp. could confer protection of heat stressed *E. coli*. In the present study, *Bacillus* was the most enriched taxa when *Ae. aegypti* were reared under increased temperatures. Understanding the mechanism that *Bacillus* spp. utilize to overcome heat stress will be very important to providing insights into the response of mosquitoes to future climate change.

Several studies have reported the impact of constant temperature increase on the life history traits of mosquitoes^40–46^. In nature, daily temperatures vary significantly and this variation likely modulates development rates^47^. Our study modelled narrower temperature ranges which are within realistic parameters for *Ae. aegypti* in nature. Our data are consistent with a number of studies demonstrating an inverse relationship between temperature and immature development^42,45,47^. The effect of temperature on the development and insect morphology has been demonstrated previously in different insects; temperature affects the relationship between wing length and body weight^48^.

Specifically, the results of our study corroborate a study carried out under field conditions at constant temperature (10–40 °C), for which, survival to adulthood and wing length were inversely related to temperature^42^. Further, although a study to compare the development time of *Ae. aegypti* under both constant temperature (12–40 °C) and natural cycling temperature demonstrated that there was no significant difference in development times^45^, Carrington et al.^47^ demonstrated that when large fluctuations in temperature are incorporated, development time of *Ae. aegypti* is accelerated compared to small fluctuations of temperature. Accelerated development equates to additional generations per year and increased opportunity for virus transmission, yet this could to some extent be offset by decreased longevity. The differences between the development time and survival in response to temperature increase suggest a tradeoff, potentially due to failure in enzyme function at high temperatures^49^.

Consistent with accelerated development, wing length decreased with an increase in temperature in this study. Wing length has been considered a good estimator of body size among Culicids^50,51^ and a direct relationship between body size and female fecundity exists^52–55^.

Better characterization of variations in mosquito fitness in response to different diurnal temperature ranges is needed to better define the role of thermal profiles in determining habitat suitability of this important disease vector.

The results of our study showed a significant dissimilarity in the midgut composition across temperature regimen. Our results are in contrast to several studies that have demonstrated a reduction in the richness of endosymbionts or complete eradication of midgut microbes in insects as a result of temperature increases^17–20^. Our studies and those studies demonstrate that temperature has an effect on gut microbes but our results may not translate to other insects sampled in other laboratories or other mosquitoes sampled in this laboratory at a different time. The dissimilarity resulting from temperature increase could significantly impact the vectorial capacity of these populations for the pathogens that they vector.

There were no significant differences in community assemblage across Miami and Mexico populations. However, richness of *Elizabethkingia* among the Mexican *Ae. aegypti* relative to the Miami *Ae. aegypti* was evident. Our previous studies demonstrate that these populations differ in their vector competence, with lower infection and transmission rates for Zika virus particularly at later time points^29^. Our findings are similar to a study on mosquito microbiome studies in the field across several sites^56–59^.

The blood meal did not significantly reduce the richness of the gut microbiota in this study, however it resulted in an altered microbial profile. Our results contrast with other studies reporting a significant alteration of the mosquito midgut microbial richness due to blood meal intake^60,61^. These differences may be due to the fact that the midgut was obtained 48 h after a blood meal; thus, the midgut microbiome describes very early stages of microbial disruption following a blood meal digestion. Indeed, microbiome have been known to fluctuate in richness throughout the mosquito life cycle^62^.

Blood meal intake by the mosquitoes resulted in the proliferation of taxa such as *Elizabethkingia*^63–65^, *Comamonadaceae*^66^*, Streptococcus*^66^*, Ralstonia*^67^ and *Chryseobacterium.* Our study corroborates the findings of^68,69^ which demonstrated that *E. anophelis* is significantly increased after a blood meal uptake, furthermore, *E. anophelis* facilitates red blood cells lysis with several hemolysins potentially contributing to blood meal digestion in mosquitoes^30,70^. Understanding the role of these taxa in the vectorial capacity and transmission efficiency of *Ae. aegypti* could lead to an improved understanding of the pathogen-insect interaction and result in the identification of novel insect control strategies and disease interventions.

## Conclusion

This study demonstrated that an increase in temperature resulted in altered life history traits and gut microbial profile of *Ae. aegypti*. An enrichment of *Bacillus*, a bacterium that has previously been shown to be associated with heat stress adaptation, was identified. Characterization of the mechanistic relationship of midgut bacteria to vector fitness and vectorial capacity is critical to characterize the impact of future climate change on *Ae. aegypti*.

## Materials and methods

### Mosquito rearing

All experimental protocols in this study were approved by Wadsworth Institutional Review Board. This study utilised two populations of *Ae. aegypti* mosquitoes [*Ae. aegypti* Miami (AEG MI) and *Ae. aegypti* Mexico (AEG MX)]. AEG MI, kindly provided by M DeGennaro, Florida International University were collected in Miami-Dade County, Florida in October 2017 and AEG MX provided by GD Ebel’s laboratory at Colorado State University (CSU) were originally collected in Poza Rica, Mexico, in 2016. These two populations were continually maintained at 28 °C with relative humidity of 60% and 16:8 h light: dark (LD) photoperiod at the New York State Department of Health (NYSDOH) Arbovirus Laboratory under standard rearing conditions.

To initiate the life history traits study, the eggs produced by colony females (F23 AEG MX and F3 AEG MI) were hatched at two diurnal temperature regimes: (H) high (day 32 °C/ night 28 °C) and (L) low (day 30 °C/ night 26 °C). Baseline temperature regimes (L) were an estimate of mean day and nighttime temperatures during peak transmission in regions from which populations were derived (www.noaa.gov) while H temperature regime modeled a 2 °C increase in baseline temperature according to the Intergovernmental Panel on Climate Change (IPCC) prediction of 2–4 °C mean global temperature rise over the next century due to global warming^71^. Temperatures were maintained in Percival environmental chambers (Perry, Iowa). To confirm the temperature and humidity of the chambers, both internal chamber log and independent HOBO data loggers (Onset, Cape Cod, MA) were used. The chambers were maintained at a relative humidity of 70–80%. Before hatching, the water was incubated for 3 h at the two different day time temperatures and the eggs were vacuum hatched in a 1 L conical flask. The vacuum hatching was in order to ensure minimal variation in hatching time. The immature stages of the mosquitoes were reared at the L and H temperature regimes.

One day post hatching, the larvae were immediately transferred to plastic rectangular flat containers [35.6 cm length × 27.9 cm width × 8.3 cm height (Sterilite, catalog no. 1963)] at a density of 200 larvae per 1 L of dechlorinated water and reared at the L and H temperature regimes at 40–60% relative humidity and a LD photoperiod 14:10. This study had three biological replicates which consisted of three different flats in each temperature regimen. Both AEG MX and AEG MI were reared at the H and L temperature regimes. Hence, the life history traits studies were calculated based on the average values. The larvae were fed 0.25 g of Tetra Pond Koi growth feed for 1st and 2nd instar larvae and 0.5 g for 3rd and 4th instar larvae.

Adults were transferred to 3.8 L cardboard cartons, held at the H and L temperature regimes and allowed to mate for 5 days while being provided with sugar and water ad libitum. Water and sugar were withdrawn 24 h before blood feed with a non-infected blood meal.

The mosquitoes were offered a blood meal twice per week, egg cups were placed in the cardboard cartons 48 h after every blood meal in order to collect eggs.

To measure the life history traits, the egg eclosion was recorded daily as well as larvae, pupae and adult survival. Larvae exuviae and cadavers were removed and counted. Larval development was recorded once daily. Due to its biological importance in pathogen transmission, upon the first day of emergence, ten females were randomly picked from each geographical origin and temperature regime. Mosquito wings were measured from the alular notch to the distal margin, excluding the fringe using Axiovision software and Zeiss microscope according to the manufacturer's specifications. The study also compared the blood meal intake across the temperature regimes, the egg clutch size and the hatch rates of the eggs produced.

### Blood meal preparation

The non-infectious blood obtained from sheep contained a final concentration of 2.5% sucrose solution was offered to the female mosquitoes after withdrawal of sugar 24 h before blood feed. All methods were carried out in accordance with relevant guidelines and regulations. The females were fed using a 37 °C pre-heated Hemotek membrane feeding system (Discovery Workshops, Acrington, UK) with a porcine sausage casing membrane. After an hour, the mosquitoes were anaesthetized with CO_2_ and immobilized on a pre-chilled tray connected to 100% CO_2._ Engorged females were separated and placed in three separate 0.6 L cardboard cartons (30 individuals per carton). Two days after the blood meal, midguts were dissected from female mosquitoes.

### DNA isolation and 16S *rRNA* gene sequencing

Amplification of the 16S *rRNA* V3-V4 hypervariable region was carried out. PCR reactions were carried out in a total volume of 50 µl, 5 µM of 16S *rRNA* primers (16S Microbiome_F TCG TCG GCA GCG TCA GAT GTG TAT AAG AGA CAG CCT ACG GGN GGC WGC AG and 16S Microbiome_R GTC TCG TGG GCT CGG AGA TCT GTA TAA GAG ACA GGA CTA CHV GGG TAT CTA ATC C), 10 µl genomic DNA and 36 µl AccuStart II PCR supermix (Quanta biosciences, Beverly, USA). The primer sequences include Illumina overhang adapter sequences integrated into them^72^. The annealing temperature of 55 °C was utilized in this PCR assay. A fragment size of ~ 460 bp of each sample was submitted to Wadsworth Center Applied Genomics Core for sequencing. Automated cluster generation and paired-end sequencing (MiSeq 500 cycle) was performed on the Illumina MiSeq system.

A total of 40 individual midguts were sequenced. Five AEG MX_L fed on 10% sucrose solution (UNFED) and five AEG MX_L fed on a non-infectious blood meal (FED), five AEG MI_L (UNFED), five AEG MI_L (FED), five AEG MX_H (UNFED), five AEG MX_H (FED), five AEG MI_H (UNFED) and five AEG MI_H (FED). A non-template control and a negative control (water) was amplified by PCR (alongside the samples) while the sequencing core spiked the sequencing run with PhiX to check for run quality.

### Statistical analysis

Statistical analysis was performed with GraphPad Prism version 5.0. The life history traits, egg hatching rates and blood feeding rates across temperature regimes was calculated using Fisher’s exact test and Chi square test. The study included three biological replicates, hence the statistical analysis of the immature stages was carried out based on the average of the three biological replicates.

Cox proportional hazard model was computed in R, the interactions between variables of geography, temperature and gender and how they affected eclosion and adult survivability were tested, ANOVA test was used to test for the statistical difference.

### Data processing and taxonomic assignment

Analysis of the data was carried out on QIITA (https://qiita.ucsd.edu/) and MicrobiomeAnalyst^73^. In summary, the command split libraries FASTQ was used to convert the de-multiplexed FASTQ files to the format used by QIITA. The samples were trimmed to 100 bp size to allow for meta-analysis. We also set a value for maximum bad run length of 3; maximum barcode errors of 1.5; Phred quality score of 33; minimum per read length fraction of 0.75. A phred quality score above 30 and above bears 0.1% chance of error.

The assignment of the reads to specific taxa was based on a 97% sequence identity to the GreenGenes 16S reference database. A BIOM-formatted OTU table was generated, subsequently. Finally, to visualize the taxonomic profiles of each sample, taxa bar plots were generated using the rarefied data artifact.

### Ecological indices and statistical analysis

Ecological indices including richness and diversity (Shannon, Bray–Curtis) were calculated using the MicrobiomeAnalyst^73^ software. Phylogenetic distance between the gut microbial community was assessed using the unweighted UniFrac distance. Indices were calculated using values from the genus taxonomic rank.

Shannon diversity is utilized to calculate alpha diversity^74^, Shannon diversity index takes into account both richness and evenness of the microbial species present. It calculates this by utilizing the proportion of species *i* relative to the total number of species (*p*_*i*_) and multiplying by the natural logarithm of the proportion (ln*p*_*i*_). Shannon Index measures the diversity of the bacterial community within an individual. On the other hand, Bray–Curtis index statistic is used to describe species counts and indicate similarity between individuals rather than difference^75^. The distance matrix generated after comparison of each sample to the other is visualized for similarity between samples using the Principle Coordinate Analysis ordination method.

To analyze for important taxa species for different experimental factors , random forest algorithm was utilized within Microbiome Analyst^73^. The default setting of the number of trees to grow and number of predictors to try was applied (500 and 7 respectively) with the randomness setting left on Random forest algorithm^76^ is a supervised classification algorithm of trees created by using bootstrap samples while training data and random feature selection in tree induction. It is an ensemble of unpruned classification or regression trees trained with the bagging method^77^.

The accession numbers of the reads sequenced in this study has been deposited in NCBI under the SRA accession numbers: PRNJA605855, PRJNA605852,PRNJA605851, PRNJA605848,PRNJA605849, PRJNA605847, PRNJA605846, PRJNA605844, PRJNA605843, PRJNA605841, PRJNA605840,PRNJA605837, PRJNA605835, PRJNA605834, PRJNA605832, PRJNA605831, PRJNA605830, PRJNA605829, PRJNA605825, PRJNA605824, PRJNA605821, PRNJA605823, PRJNA605819, PRJNA605806, PRJNA605793, PRNJA605788, PRJNA605792, PRJNA605779, PRJNA605785, PRJNA605787, PRJNA605772, PRJNA605773, PRJNA605770, PRJNA605767, PRJNA605764,PRNJA605763, PRJNA605750, PRJNA605761, PRJNA605747.

## Supplementary information

Supplementary File 1.

Supplementary File 2.

Supplementary File 3.

Supplementary Legends.

## Data Availability

The 16S rRNA reads is available at the NCBI SRA portal https://www.ncbi.nlm.nih.gov/sra.
