# Supplementary material for: Increase in temperature enriches heat tolerant taxa in Aedes aegypti midguts
Source: Sci Rep. 2020 Nov 5;10:19135. doi: 10.1038/s41598-020-76188-x (PMC7644690; doi:10.1038/s41598-020-76188-x)
Supplement: Supplementary file 4 — Supplementary Legends. [file 41598_2020_76188_MOESM4_ESM.docx]

**Supplementary files**

Supplementary file no. 1. A cox proportional hazard model R code for the eclosion model

Supplementary file no. 2. A cox proportional hazard model R code for the survival model

Supplementary file no.3. An excel file representing the taxa identified in the midguts of *Aedes aegypti* of different blood feed status and reared at increased temperatures.
